# Supplementary material for: The relationship between anti-Müllerian hormone (AMH) levels and pregnancy outcomes in patients undergoing assisted reproductive techniques (ART)
Source: PeerJ. 2020 Dec 22;8:e10390. doi: 10.7717/peerj.10390 (PMC7761264; doi:10.7717/peerj.10390)
Supplement: Supplemental Information 1 [file peerj-08-10390-s001.zip › Raw data/Correlation frequencies.docx]

| **AMHcat** | | | | | |
| --- | --- | --- | --- | --- | --- |
|  | | Frequency | Percent | Valid Percent | Cumulative Percent |
| Valid | 0.5 - 1.0 | 4 | 8.0 | 8.2 | 8.2 |
|  | 1.01 -1.50 | 10 | 20.0 | 20.4 | 28.6 |
|  | 1.501 - 4.0 | 22 | 44.0 | 44.9 | 73.5 |
|  | >1.40 | 13 | 26.0 | 26.5 | 100.0 |
|  | Total | 49 | 98.0 | 100.0 |  |
| Missing | System | 1 | 2.0 |  |  |
| Total | | 50 | 100.0 |  |  |

| **no_mature_cat** | | | | | |
| --- | --- | --- | --- | --- | --- |
|  | | Frequency | Percent | Valid Percent | Cumulative Percent |
| Valid | .00 | 4 | 8.0 | 8.0 | 8.0 |
|  | 1.00 | 7 | 14.0 | 14.0 | 22.0 |
|  | 2.00 | 18 | 36.0 | 36.0 | 58.0 |
|  | 3.00 | 11 | 22.0 | 22.0 | 80.0 |
|  | >=4.00 | 10 | 20.0 | 20.0 | 100.0 |
|  | Total | 50 | 100.0 | 100.0 |  |

**Frequency Table**

| **Race** | | | | | |
| --- | --- | --- | --- | --- | --- |
|  | | Frequency | Percent | Valid Percent | Cumulative Percent |
| Valid | Black | 11 | 22.0 | 22.0 | 22.0 |
|  | Colored | 4 | 8.0 | 8.0 | 30.0 |
|  | Indian | 26 | 52.0 | 52.0 | 82.0 |
|  | White | 9 | 18.0 | 18.0 | 100.0 |
|  | Total | 50 | 100.0 | 100.0 |  |

| **No_of_oocytes** | | | | | |
| --- | --- | --- | --- | --- | --- |
|  | | Frequency | Percent | Valid Percent | Cumulative Percent |
| Valid | 1 | 7 | 14.0 | 14.0 | 14.0 |
|  | 2 | 20 | 40.0 | 40.0 | 54.0 |
|  | 3 | 11 | 22.0 | 22.0 | 76.0 |
|  | 4 | 7 | 14.0 | 14.0 | 90.0 |
|  | 5 | 3 | 6.0 | 6.0 | 96.0 |
|  | 9 | 1 | 2.0 | 2.0 | 98.0 |
|  | 11 | 1 | 2.0 | 2.0 | 100.0 |
|  | Total | 50 | 100.0 | 100.0 |  |

| **No_mature** | | | | | |
| --- | --- | --- | --- | --- | --- |
|  | | Frequency | Percent | Valid Percent | Cumulative Percent |
| Valid | 0 | 4 | 8.0 | 8.0 | 8.0 |
|  | 1 | 7 | 14.0 | 14.0 | 22.0 |
|  | 2 | 18 | 36.0 | 36.0 | 58.0 |
|  | 3 | 11 | 22.0 | 22.0 | 80.0 |
|  | 4 | 7 | 14.0 | 14.0 | 94.0 |
|  | 5 | 1 | 2.0 | 2.0 | 96.0 |
|  | 6 | 1 | 2.0 | 2.0 | 98.0 |
|  | 7 | 1 | 2.0 | 2.0 | 100.0 |
|  | Total | 50 | 100.0 | 100.0 |  |

| **No_Fertilized** | | | | | |
| --- | --- | --- | --- | --- | --- |
|  | | Frequency | Percent | Valid Percent | Cumulative Percent |
| Valid | 1 | 8 | 16.0 | 16.0 | 16.0 |
|  | 2 | 20 | 40.0 | 40.0 | 56.0 |
|  | 3 | 13 | 26.0 | 26.0 | 82.0 |
|  | 4 | 6 | 12.0 | 12.0 | 94.0 |
|  | 5 | 1 | 2.0 | 2.0 | 96.0 |
|  | 6 | 1 | 2.0 | 2.0 | 98.0 |
|  | 7 | 1 | 2.0 | 2.0 | 100.0 |
|  | Total | 50 | 100.0 | 100.0 |  |

| **No _transfer** | | | | | |
| --- | --- | --- | --- | --- | --- |
|  | | Frequency | Percent | Valid Percent | Cumulative Percent |
| Valid |  | 2 | 4.0 | 4.0 | 4.0 |
|  | 1 | 9 | 18.0 | 18.0 | 22.0 |
|  | 2 | 28 | 56.0 | 56.0 | 78.0 |
|  | 3 | 3 | 6.0 | 6.0 | 84.0 |
|  | Abnormal | 8 | 16.0 | 16.0 | 100.0 |
|  | Total | 50 | 100.0 | 100.0 |  |

| **Day_Transfer** | | | | | |
| --- | --- | --- | --- | --- | --- |
|  | | Frequency | Percent | Valid Percent | Cumulative Percent |
| Valid |  | 8 | 16.0 | 16.0 | 16.0 |
|  | Day 3 | 3 | 6.0 | 6.0 | 22.0 |
|  | Day 4 | 3 | 6.0 | 6.0 | 28.0 |
|  | Day 5 | 30 | 60.0 | 60.0 | 88.0 |
|  | Day 6 | 6 | 12.0 | 12.0 | 100.0 |
|  | Total | 50 | 100.0 | 100.0 |  |

| **Result** | | | | | |
| --- | --- | --- | --- | --- | --- |
|  | | Frequency | Percent | Valid Percent | Cumulative Percent |
| Valid |  | 8 | 16.0 | 16.0 | 16.0 |
|  | Neg | 30 | 60.0 | 60.0 | 76.0 |
|  | POS | 12 | 24.0 | 24.0 | 100.0 |
|  | Total | 50 | 100.0 | 100.0 |  |

| **AMHcat** | | | | | |
| --- | --- | --- | --- | --- | --- |
|  | | Frequency | Percent | Valid Percent | Cumulative Percent |
| Valid | 0.5 - 1.0 | 4 | 8.0 | 8.2 | 8.2 |
|  | 1.01 -1.50 | 10 | 20.0 | 20.4 | 28.6 |
|  | 1.501 - 4.0 | 22 | 44.0 | 44.9 | 73.5 |
|  | >1.40 | 13 | 26.0 | 26.5 | 100.0 |
|  | Total | 49 | 98.0 | 100.0 |  |
| Missing | System | 1 | 2.0 |  |  |
| Total | | 50 | 100.0 |  |  |

| **no_mature_cat** | | | | | |
| --- | --- | --- | --- | --- | --- |
|  | | Frequency | Percent | Valid Percent | Cumulative Percent |
| Valid | .00 | 4 | 8.0 | 8.0 | 8.0 |
|  | 1.00 | 7 | 14.0 | 14.0 | 22.0 |
|  | 2.00 | 18 | 36.0 | 36.0 | 58.0 |
|  | 3.00 | 11 | 22.0 | 22.0 | 80.0 |
|  | 4.00 | 10 | 20.0 | 20.0 | 100.0 |
|  | Total | 50 | 100.0 | 100.0 |  |

| **AMH2cat** | | | | | |
| --- | --- | --- | --- | --- | --- |
|  | | Frequency | Percent | Valid Percent | Cumulative Percent |
| Valid | 0.5 - 1.5 | 14 | 28.0 | 28.6 | 28.6 |
|  | >1.5 | 35 | 70.0 | 71.4 | 100.0 |
|  | Total | 49 | 98.0 | 100.0 |  |
| Missing | System | 1 | 2.0 |  |  |
| Total | | 50 | 100.0 |  |  |
