# Supplementary material for: The relationship between anti-Müllerian hormone (AMH) levels and pregnancy outcomes in patients undergoing assisted reproductive techniques (ART)
Source: PeerJ. 2020 Dec 22;8:e10390. doi: 10.7717/peerj.10390 (PMC7761264; doi:10.7717/peerj.10390)
Supplement: Supplemental Information 1 [file peerj-08-10390-s001.zip › Raw data/correlations AMH FSH AGE OOCYTES.docx]

**Correlations: AMH FSH age and No oocytes**

| **Correlations** | | | | | |
| --- | --- | --- | --- | --- | --- |
|  | | BasalFSH | BasalAMH | Age | No_of_oocytes |
| BasalFSH | Pearson Correlation | 1 | -.185 | -.087 | .179 |
|  | Sig. (2-tailed) |  | .240 | .583 | .256 |
|  | N | 42 | 42 | 42 | 42 |
| BasalAMH | Pearson Correlation | -.185 | 1 | -.028 | .206 |
|  | Sig. (2-tailed) | .240 |  | .859 | .191 |
|  | N | 42 | 42 | 42 | 42 |
| Age | Pearson Correlation | -.087 | -.028 | 1 | -.271 |
|  | Sig. (2-tailed) | .583 | .859 |  | .082 |
|  | N | 42 | 42 | 42 | 42 |
| No_of_oocytes | Pearson Correlation | .179 | .206 | -.271 | 1 |
|  | Sig. (2-tailed) | .256 | .191 | .082 |  |
|  | N | 42 | 42 | 42 | 42 |
