# Supplementary material for: The relationship between anti-Müllerian hormone (AMH) levels and pregnancy outcomes in patients undergoing assisted reproductive techniques (ART)
Source: PeerJ. 2020 Dec 22;8:e10390. doi: 10.7717/peerj.10390 (PMC7761264; doi:10.7717/peerj.10390)

**Correlations**


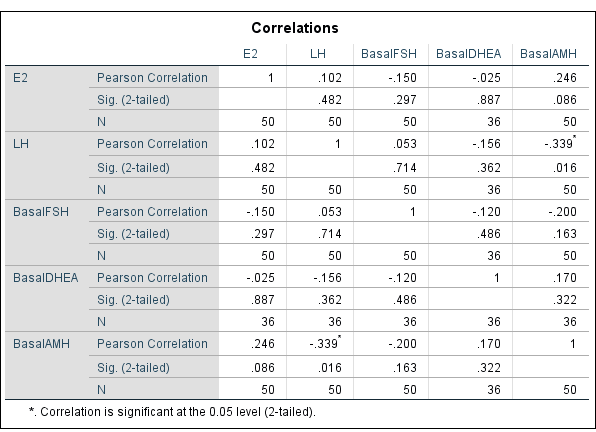


**Logistic regression model**

| **Variables in the Equation** | | | | | | | |
| --- | --- | --- | --- | --- | --- | --- | --- |
|  | | B | S.E. | Wald | df | Sig. | Exp(B) |
| Step 1^a^ | Age | -.096 | .083 | 1.357 | 1 | .244 | .908 |
|  | BasalAMH | -.087 | .191 | .205 | 1 | .651 | .917 |
|  | No_mature | .463 | .260 | 3.171 | 1 | .075 | 1.589 |
|  | Constant | 1.330 | 2.800 | .226 | 1 | .635 | 3.783 |
| a. Variable(s) entered on step 1: Age, BasalAMH, No_mature. | | | | | | | |

With race but not significant

| **Variables in the Equation** | | | | | | | | | |
| --- | --- | --- | --- | --- | --- | --- | --- | --- | --- |
|  | | B | S.E. | Wald | df | Sig. | Exp(B) | 95% C.I.for EXP(B) | |
|  |  |  |  |  |  |  |  | Lower | Upper |
| Step 1^a^ | Age | -.089 | .088 | 1.041 | 1 | .308 | .915 | .770 | 1.086 |
|  | BasalAMH | -.141 | .225 | .397 | 1 | .529 | .868 | .559 | 1.348 |
|  | No_mature | .468 | .267 | 3.086 | 1 | .079 | 1.597 | .947 | 2.693 |
|  | Race |  |  | 1.844 | 3 | .605 |  |  |  |
|  | Race(1) | .199 | 1.158 | .029 | 1 | .864 | 1.220 | .126 | 11.813 |
|  | Race(2) | .902 | 1.412 | .408 | 1 | .523 | 2.464 | .155 | 39.211 |
|  | Race(3) | -.658 | .973 | .457 | 1 | .499 | .518 | .077 | 3.487 |
|  | Constant | 1.431 | 3.104 | .213 | 1 | .645 | 4.184 |  |  |
| a. Variable(s) entered on step 1: Age, BasalAMH, No_mature, Race. | | | | | | | | | |

Another model with age E2 LH AMH

| **Variables in the Equation** | | | | | | | | | |
| --- | --- | --- | --- | --- | --- | --- | --- | --- | --- |
|  | | B | S.E. | Wald | df | Sig. | Exp(B) | 95% C.I.for EXP(B) | |
|  |  |  |  |  |  |  |  | Lower | Upper |
| Step 1^a^ | Age | -.142 | .091 | 2.451 | 1 | .117 | .867 | .726 | 1.037 |
|  | No_mature | .261 | .304 | .737 | 1 | .390 | 1.298 | .716 | 2.355 |
|  | E2 | .001 | .000 | 3.580 | 1 | .058 | 1.001 | 1.000 | 1.002 |
|  | LH | -.600 | .303 | 3.923 | 1 | .048 | .549 | .303 | .994 |
|  | BasalAMH | -.309 | .229 | 1.821 | 1 | .177 | .734 | .469 | 1.150 |
|  | Constant | 3.333 | 3.156 | 1.116 | 1 | .291 | 28.031 |  |  |
| a. Variable(s) entered on step 1: Age, No_mature, E2, LH, BasalAMH. | | | | | | | | | |

ROC Area under the curve.

Classification categories

- .90-1 = excellent (A)
- .80-.90 = good (B)
- .70-.80 = fair (C)
- .60-.70 = poor (D)
- .50-.60 = fail (F)

AREA UNDER THE CURVE FOR E2: AUC =0.725 (fair)


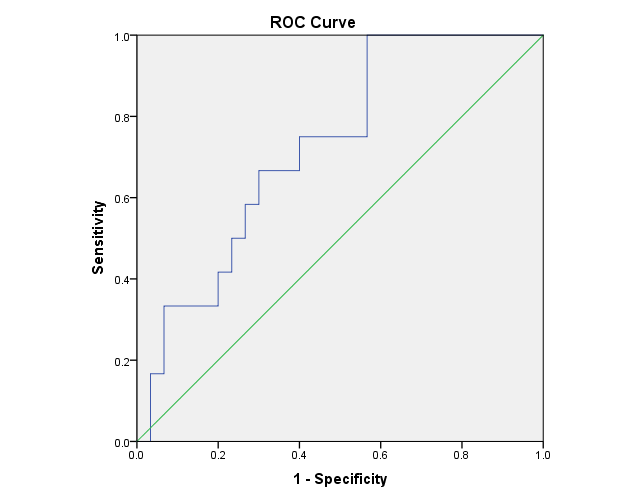

Supplement: Supplemental Information 1 [file peerj-08-10390-s001.zip › Raw data/Correlations and logistic regression.docx]
