# Supplementary material for: The relationship between anti-Müllerian hormone (AMH) levels and pregnancy outcomes in patients undergoing assisted reproductive techniques (ART)
Source: PeerJ. 2020 Dec 22;8:e10390. doi: 10.7717/peerj.10390 (PMC7761264; doi:10.7717/peerj.10390)
Supplement: Supplemental Information 1 [file peerj-08-10390-s001.zip › Raw data/Cross tabs etc.docx]

**Frequencies**

|  |
| --- |

| **Result** | | | | | |
| --- | --- | --- | --- | --- | --- |
|  | | Frequency | Percent | Valid Percent | Cumulative Percent |
| Valid | Neg | 30 | 71.4 | 71.4 | 71.4 |
|  | POS | 12 | 28.6 | 28.6 | 100.0 |
|  | Total | 42 | 100.0 | 100.0 |  |

**Logistic Regression**

| **Case Processing Summary** | | | |
| --- | --- | --- | --- |
| Unweighted Cases^a^ | | N | Percent |
| Selected Cases | Included in Analysis | 42 | 100.0 |
|  | Missing Cases | 0 | .0 |
|  | Total | 42 | 100.0 |
| Unselected Cases | | 0 | .0 |
| Total | | 42 | 100.0 |
| a. If weight is in effect, see classification table for the total number of cases. | | | |

| **Dependent Variable Encoding** | |
| --- | --- |
| Original Value | Internal Value |
| .00 | 0 |
| 1.00 | 1 |

**Block 0: Beginning Block**

| **Classification Table^a,b^** | | | | | |
| --- | --- | --- | --- | --- | --- |
|  | Observed | | Predicted | | |
|  |  |  | outcome | | Percentage Correct |
|  |  |  | .00 | 1.00 |  |
| Step 0 | outcome | .00 | 30 | 0 | 100.0 |
|  |  | 1.00 | 12 | 0 | .0 |
|  | Overall Percentage | |  |  | 71.4 |
| a. Constant is included in the model. | | | | | |
| b. The cut value is .500 | | | | | |

| **Variables in the Equation** | | | | | | | |
| --- | --- | --- | --- | --- | --- | --- | --- |
|  | | B | S.E. | Wald | df | Sig. | Exp(B) |
| Step 0 | Constant | -.916 | .342 | 7.196 | 1 | .007 | .400 |

| **Variables not in the Equation** | | | | | |
| --- | --- | --- | --- | --- | --- |
|  | | | Score | df | Sig. |
| Step 0 | Variables | E2 | 3.985 | 1 | .046 |
|  |  | LH | 1.400 | 1 | .237 |
|  |  | No_of_oocytes | 2.280 | 1 | .131 |
|  |  | BasalFSH | .735 | 1 | .391 |
|  |  | BasalAMH | .016 | 1 | .900 |
|  | Overall Statistics | | 9.699 | 5 | .084 |

**Block 1: Method = Enter**

| **Omnibus Tests of Model Coefficients** | | | | |
| --- | --- | --- | --- | --- |
|  | | Chi-square | df | Sig. |
| Step 1 | Step | 10.976 | 5 | .052 |
|  | Block | 10.976 | 5 | .052 |
|  | Model | 10.976 | 5 | .052 |

| **Model Summary** | | | |
| --- | --- | --- | --- |
| Step | -2 Log likelihood | Cox & Snell R Square | Nagelkerke R Square |
| 1 | 39.279^a^ | .230 | .330 |
| a. Estimation terminated at iteration number 5 because parameter estimates changed by less than .001. | | | |

| **Classification Table^a^** | | | | | |
| --- | --- | --- | --- | --- | --- |
|  | Observed | | Predicted | | |
|  |  |  | outcome | | Percentage Correct |
|  |  |  | .00 | 1.00 |  |
| Step 1 | outcome | .00 | 26 | 4 | 86.7 |
|  |  | 1.00 | 8 | 4 | 33.3 |
|  | Overall Percentage | |  |  | 71.4 |
| a. The cut value is .500 | | | | | |

| **Variables in the Equation** | | | | | | | |
| --- | --- | --- | --- | --- | --- | --- | --- |
|  | | B | S.E. | Wald | df | Sig. | Exp(B) |
| Step 1^a^ | E2 | .001 | .000 | 2.589 | 1 | .108 | 1.001 |
|  | LH | -.498 | .261 | 3.655 | 1 | .056 | .608 |
|  | No_of_oocytes | .268 | .240 | 1.250 | 1 | .264 | 1.307 |
|  | BasalFSH | -.119 | .134 | .789 | 1 | .374 | .888 |
|  | BasalAMH | -.334 | .230 | 2.110 | 1 | .146 | .716 |
|  | Constant | -.273 | 1.499 | .033 | 1 | .856 | .761 |
| a. Variable(s) entered on step 1: E2, LH, No_of_oocytes, BasalFSH, BasalAMH. | | | | | | | |

**Logistic Regression**

| **Dependent Variable Encoding** | |
| --- | --- |
| Original Value | Internal Value |
| .00 | 0 |
| 1.00 | 1 |

**Block 0: Beginning Block**

| **Classification Table^a,b^** | | | | | |
| --- | --- | --- | --- | --- | --- |
|  | Observed | | Predicted | | |
|  |  |  | outcome | | Percentage Correct |
|  |  |  | .00 | 1.00 |  |
| Step 0 | outcome | .00 | 30 | 0 | 100.0 |
|  |  | 1.00 | 12 | 0 | .0 |
|  | Overall Percentage | |  |  | 71.4 |
| a. Constant is included in the model. | | | | | |
| b. The cut value is .500 | | | | | |

| **Variables in the Equation** | | | | | | | |
| --- | --- | --- | --- | --- | --- | --- | --- |
|  | | B | S.E. | Wald | df | Sig. | Exp(B) |
| Step 0 | Constant | -.916 | .342 | 7.196 | 1 | .007 | .400 |

| **Variables not in the Equation** | | | | | |
| --- | --- | --- | --- | --- | --- |
|  | | | Score | df | Sig. |
| Step 0 | Variables | E2 | 3.985 | 1 | .046 |
|  |  | LH | 1.400 | 1 | .237 |
|  |  | No_of_oocytes | 2.280 | 1 | .131 |
|  |  | BasalFSH | .735 | 1 | .391 |
|  |  | BasalAMH | .016 | 1 | .900 |
|  |  | Age | 1.760 | 1 | .185 |
|  | Overall Statistics | | 10.869 | 6 | .093 |

**Block 1: Method = Enter**

| **Omnibus Tests of Model Coefficients** | | | | |
| --- | --- | --- | --- | --- |
|  | | Chi-square | df | Sig. |
| Step 1 | Step | 13.360 | 6 | .038 |
|  | Block | 13.360 | 6 | .038 |
|  | Model | 13.360 | 6 | .038 |

| **Model Summary** | | | |
| --- | --- | --- | --- |
| Step | -2 Log likelihood | Cox & Snell R Square | Nagelkerke R Square |
| 1 | 36.895^a^ | .272 | .390 |
| a. Estimation terminated at iteration number 6 because parameter estimates changed by less than .001. | | | |

| **Classification Table^a^** | | | | | |
| --- | --- | --- | --- | --- | --- |
|  | Observed | | Predicted | | |
|  |  |  | outcome | | Percentage Correct |
|  |  |  | .00 | 1.00 |  |
| Step 1 | outcome | .00 | 25 | 5 | 83.3 |
|  |  | 1.00 | 7 | 5 | 41.7 |
|  | Overall Percentage | |  |  | 71.4 |
| a. The cut value is .500 | | | | | |

| **Variables in the Equation** | | | | | | | |
| --- | --- | --- | --- | --- | --- | --- | --- |
|  | | B | S.E. | Wald | df | Sig. | Exp(B) |
| Step 1^a^ | E2 | .001 | .000 | 3.161 | 1 | .075 | 1.001 |
|  | LH | -.591 | .296 | 3.978 | 1 | .046 | .554 |
|  | No_of_oocytes | .194 | .240 | .652 | 1 | .419 | 1.214 |
|  | BasalFSH | -.129 | .145 | .785 | 1 | .376 | .879 |
|  | BasalAMH | -.360 | .241 | 2.236 | 1 | .135 | .698 |
|  | Age | -.135 | .091 | 2.185 | 1 | .139 | .874 |
|  | Constant | 4.355 | 3.457 | 1.587 | 1 | .208 | 77.852 |
| a. Variable(s) entered on step 1: E2, LH, No_of_oocytes, BasalFSH, BasalAMH, Age. | | | | | | | |

LOGISTIC REGRESSION VARIABLES outcome

/METHOD=ENTER E2 LH BasalAMH Age

/CRITERIA=PIN(.05) POUT(.10) ITERATE(20) CUT(.5).

**Logistic Regression**

| **Case Processing Summary** | | | |
| --- | --- | --- | --- |
| Unweighted Cases^a^ | | N | Percent |
| Selected Cases | Included in Analysis | 42 | 100.0 |
|  | Missing Cases | 0 | .0 |
|  | Total | 42 | 100.0 |
| Unselected Cases | | 0 | .0 |
| Total | | 42 | 100.0 |
| a. If weight is in effect, see classification table for the total number of cases. | | | |

| **Dependent Variable Encoding** | |
| --- | --- |
| Original Value | Internal Value |
| .00 | 0 |
| 1.00 | 1 |

**Block 0: Beginning Block**

| **Classification Table^a,b^** | | | | | |
| --- | --- | --- | --- | --- | --- |
|  | Observed | | Predicted | | |
|  |  |  | outcome | | Percentage Correct |
|  |  |  | .00 | 1.00 |  |
| Step 0 | outcome | .00 | 30 | 0 | 100.0 |
|  |  | 1.00 | 12 | 0 | .0 |
|  | Overall Percentage | |  |  | 71.4 |
| a. Constant is included in the model. | | | | | |
| b. The cut value is .500 | | | | | |

| **Variables in the Equation** | | | | | | | |
| --- | --- | --- | --- | --- | --- | --- | --- |
|  | | B | S.E. | Wald | df | Sig. | Exp(B) |
| Step 0 | Constant | -.916 | .342 | 7.196 | 1 | .007 | .400 |

| **Variables not in the Equation** | | | | | |
| --- | --- | --- | --- | --- | --- |
|  | | | Score | df | Sig. |
| Step 0 | Variables | E2 | 3.985 | 1 | .046 |
|  |  | LH | 1.400 | 1 | .237 |
|  |  | BasalAMH | .016 | 1 | .900 |
|  |  | Age | 1.760 | 1 | .185 |
|  | Overall Statistics | | 9.453 | 4 | .051 |

**Block 1: Method = Enter**

| **Omnibus Tests of Model Coefficients** | | | | |
| --- | --- | --- | --- | --- |
|  | | Chi-square | df | Sig. |
| Step 1 | Step | 12.202 | 4 | .016 |
|  | Block | 12.202 | 4 | .016 |
|  | Model | 12.202 | 4 | .016 |

| **Model Summary** | | | |
| --- | --- | --- | --- |
| Step | -2 Log likelihood | Cox & Snell R Square | Nagelkerke R Square |
| 1 | 38.053^a^ | .252 | .361 |
| a. Estimation terminated at iteration number 6 because parameter estimates changed by less than .001. | | | |

| **Classification Table^a^** | | | | | |
| --- | --- | --- | --- | --- | --- |
|  | Observed | | Predicted | | |
|  |  |  | outcome | | Percentage Correct |
|  |  |  | .00 | 1.00 |  |
| Step 1 | outcome | .00 | 25 | 5 | 83.3 |
|  |  | 1.00 | 7 | 5 | 41.7 |
|  | Overall Percentage | |  |  | 71.4 |
| a. The cut value is .500 | | | | | |

| **Variables in the Equation** | | | | | | | |
| --- | --- | --- | --- | --- | --- | --- | --- |
|  | | B | S.E. | Wald | df | Sig. | Exp(B) |
| Step 1^a^ | E2 | .001 | .000 | 5.672 | 1 | .017 | 1.001 |
|  | LH | -.571 | .271 | 4.450 | 1 | .035 | .565 |
|  | BasalAMH | -.284 | .224 | 1.607 | 1 | .205 | .753 |
|  | Age | -.146 | .090 | 2.624 | 1 | .105 | .864 |
|  | Constant | 3.680 | 3.100 | 1.409 | 1 | .235 | 39.662 |
| a. Variable(s) entered on step 1: E2, LH, BasalAMH, Age. | | | | | | | |

LOGISTIC REGRESSION VARIABLES outcome

/METHOD=ENTER E2 LH BasalAMH Age No_Fertilized

/CRITERIA=PIN(.05) POUT(.10) ITERATE(20) CUT(.5).

**Logistic Regression**

| **Dependent Variable Encoding** | |
| --- | --- |
| Original Value | Internal Value |
| .00 | 0 |
| 1.00 | 1 |

**Block 0: Beginning Block**

| **Classification Table^a,b^** | | | | | |
| --- | --- | --- | --- | --- | --- |
|  | Observed | | Predicted | | |
|  |  |  | outcome | | Percentage Correct |
|  |  |  | .00 | 1.00 |  |
| Step 0 | outcome | .00 | 30 | 0 | 100.0 |
|  |  | 1.00 | 12 | 0 | .0 |
|  | Overall Percentage | |  |  | 71.4 |
| a. Constant is included in the model. | | | | | |
| b. The cut value is .500 | | | | | |

| **Variables in the Equation** | | | | | | | |
| --- | --- | --- | --- | --- | --- | --- | --- |
|  | | B | S.E. | Wald | df | Sig. | Exp(B) |
| Step 0 | Constant | -.916 | .342 | 7.196 | 1 | .007 | .400 |

| **Variables not in the Equation** | | | | | |
| --- | --- | --- | --- | --- | --- |
|  | | | Score | df | Sig. |
| Step 0 | Variables | E2 | 3.985 | 1 | .046 |
|  |  | LH | 1.400 | 1 | .237 |
|  |  | BasalAMH | .016 | 1 | .900 |
|  |  | Age | 1.760 | 1 | .185 |
|  |  | No_Fertilized | 2.473 | 1 | .116 |
|  | Overall Statistics | | 9.453 | 5 | .092 |

**Block 1: Method = Enter**

| **Omnibus Tests of Model Coefficients** | | | | |
| --- | --- | --- | --- | --- |
|  | | Chi-square | df | Sig. |
| Step 1 | Step | 12.248 | 5 | .032 |
|  | Block | 12.248 | 5 | .032 |
|  | Model | 12.248 | 5 | .032 |

| **Model Summary** | | | |
| --- | --- | --- | --- |
| Step | -2 Log likelihood | Cox & Snell R Square | Nagelkerke R Square |
| 1 | 38.007^a^ | .253 | .363 |
| a. Estimation terminated at iteration number 6 because parameter estimates changed by less than .001. | | | |

| **Classification Table^a^** | | | | | |
| --- | --- | --- | --- | --- | --- |
|  | Observed | | Predicted | | |
|  |  |  | outcome | | Percentage Correct |
|  |  |  | .00 | 1.00 |  |
| Step 1 | outcome | .00 | 25 | 5 | 83.3 |
|  |  | 1.00 | 8 | 4 | 33.3 |
|  | Overall Percentage | |  |  | 69.0 |
| a. The cut value is .500 | | | | | |

| **Variables in the Equation** | | | | | | | |
| --- | --- | --- | --- | --- | --- | --- | --- |
|  | | B | S.E. | Wald | df | Sig. | Exp(B) |
| Step 1^a^ | E2 | .001 | .000 | 4.532 | 1 | .033 | 1.001 |
|  | LH | -.576 | .278 | 4.289 | 1 | .038 | .562 |
|  | BasalAMH | -.292 | .227 | 1.646 | 1 | .200 | .747 |
|  | Age | -.144 | .091 | 2.519 | 1 | .113 | .866 |
|  | No_Fertilized | .076 | .354 | .046 | 1 | .830 | 1.079 |
|  | Constant | 3.516 | 3.190 | 1.215 | 1 | .270 | 33.644 |
| a. Variable(s) entered on step 1: E2, LH, BasalAMH, Age, No_Fertilized. | | | | | | | |

**Logistic Regression**

| **Case Processing Summary** | | | |
| --- | --- | --- | --- |
| Unweighted Cases^a^ | | N | Percent |
| Selected Cases | Included in Analysis | 42 | 100.0 |
|  | Missing Cases | 0 | .0 |
|  | Total | 42 | 100.0 |
| Unselected Cases | | 0 | .0 |
| Total | | 42 | 100.0 |
| a. If weight is in effect, see classification table for the total number of cases. | | | |

| **Dependent Variable Encoding** | |
| --- | --- |
| Original Value | Internal Value |
| .00 | 0 |
| 1.00 | 1 |

**Block 0: Beginning Block**

| **Classification Table^a,b^** | | | | | |
| --- | --- | --- | --- | --- | --- |
|  | Observed | | Predicted | | |
|  |  |  | outcome | | Percentage Correct |
|  |  |  | .00 | 1.00 |  |
| Step 0 | outcome | .00 | 30 | 0 | 100.0 |
|  |  | 1.00 | 12 | 0 | .0 |
|  | Overall Percentage | |  |  | 71.4 |
| a. Constant is included in the model. | | | | | |
| b. The cut value is .500 | | | | | |

| **Variables in the Equation** | | | | | | | |
| --- | --- | --- | --- | --- | --- | --- | --- |
|  | | B | S.E. | Wald | df | Sig. | Exp(B) |
| Step 0 | Constant | -.916 | .342 | 7.196 | 1 | .007 | .400 |

| **Variables not in the Equation** | | | | | |
| --- | --- | --- | --- | --- | --- |
|  | | | Score | df | Sig. |
| Step 0 | Variables | E2 | 3.985 | 1 | .046 |
|  |  | LH | 1.400 | 1 | .237 |
|  |  | BasalAMH | .016 | 1 | .900 |
|  |  | Age | 1.760 | 1 | .185 |
|  |  | No_of_oocytes | 2.280 | 1 | .131 |
|  | Overall Statistics | | 9.580 | 5 | .088 |

**Block 1: Method = Enter**

| **Omnibus Tests of Model Coefficients** | | | | |
| --- | --- | --- | --- | --- |
|  | | Chi-square | df | Sig. |
| Step 1 | Step | 12.493 | 5 | .029 |
|  | Block | 12.493 | 5 | .029 |
|  | Model | 12.493 | 5 | .029 |

| **Model Summary** | | | |
| --- | --- | --- | --- |
| Step | -2 Log likelihood | Cox & Snell R Square | Nagelkerke R Square |
| 1 | 37.761^a^ | .257 | .369 |
| a. Estimation terminated at iteration number 6 because parameter estimates changed by less than .001. | | | |

| **Classification Table^a^** | | | | | |
| --- | --- | --- | --- | --- | --- |
|  | Observed | | Predicted | | |
|  |  |  | outcome | | Percentage Correct |
|  |  |  | .00 | 1.00 |  |
| Step 1 | outcome | .00 | 26 | 4 | 86.7 |
|  |  | 1.00 | 8 | 4 | 33.3 |
|  | Overall Percentage | |  |  | 71.4 |
| a. The cut value is .500 | | | | | |

| **Variables in the Equation** | | | | | | | |
| --- | --- | --- | --- | --- | --- | --- | --- |
|  | | B | S.E. | Wald | df | Sig. | Exp(B) |
| Step 1^a^ | E2 | .001 | .000 | 4.583 | 1 | .032 | 1.001 |
|  | LH | -.604 | .299 | 4.067 | 1 | .044 | .547 |
|  | BasalAMH | -.304 | .227 | 1.788 | 1 | .181 | .738 |
|  | Age | -.136 | .092 | 2.202 | 1 | .138 | .873 |
|  | No_of_oocytes | .119 | .223 | .284 | 1 | .594 | 1.126 |
|  | Constant | 3.255 | 3.194 | 1.039 | 1 | .308 | 25.924 |
| a. Variable(s) entered on step 1: E2, LH, BasalAMH, Age, No_of_oocytes. | | | | | | | |

FREQUENCIES VARIABLES=newAMHcat

/ORDER=ANALYSIS.

**Frequencies**

| **Statistics** | | |
| --- | --- | --- |
| newAMHcat | | |
| N | Valid | 42 |
|  | Missing | 0 |

FREQUENCIES VARIABLES=newAMHcat AMH3cat

/ORDER=ANALYSIS.

**Frequency Table**

**Crosstabs**

| **newAMHcat * Day_Transfer Crosstabulation** | | | | | | |
| --- | --- | --- | --- | --- | --- | --- |
| Count | | | | | | |
|  | | Day_Transfer | | | | Total |
|  |  | Day 3 | Day 4 | Day 5 | Day 6 |  |
| newAMHcat | 3.00 | 0 | 1 | 0 | 0 | 1 |
|  | 4.00 | 1 | 1 | 15 | 2 | 19 |
|  | 5.00 | 2 | 1 | 15 | 4 | 22 |
| Total | | 3 | 3 | 30 | 6 | 42 |

| **Chi-Square Tests** | | | |
| --- | --- | --- | --- |
|  | Value | df | Asymptotic Significance (2-sided) |
| Pearson Chi-Square | 14.117^a^ | 6 | .028 |
| Likelihood Ratio | 6.432 | 6 | .377 |
| N of Valid Cases | 42 |  |  |
| a. 10 cells (83.3%) have expected count less than 5. The minimum expected count is .07. | | | |

**Crosstabs**

| **Case Processing Summary** | | | | | | | | | |  |  |
| --- | --- | --- | --- | --- | --- | --- | --- | --- | --- | --- | --- |
|  | | | Cases | | | | | | |  |  |
| **newAMHcat * No_Fertilized Crosstabulation** | | | | | | | | | | | |
| Count | | | | | | | | | | | |
|  | | No_Fertilized | | | | | | | | | Total |
|  |  | 1 | | 2 | 3 | 4 | 5 | 6 | 7 | |  |
| newAMHcat | 3.00 | 1 | | 0 | 0 | 0 | 0 | 0 | 0 | | 1 |
|  | 4.00 | 2 | | 13 | 2 | 2 | 0 | 0 | 0 | | 19 |
|  | 5.00 | 3 | | 4 | 8 | 4 | 1 | 1 | 1 | | 22 |
| Total | | 6 | | 17 | 10 | 6 | 1 | 1 | 1 | | 42 |

| **Chi-Square Tests** | | | | | | | | |  |  |
| --- | --- | --- | --- | --- | --- | --- | --- | --- | --- | --- |
|  | | | Value | | df | | Asymptotic Significance (2-sided) | |  |  |
| Pearson Chi-Square | | | 18.504^a^ | | 12 | | .101 | |  |  |
| Likelihood Ratio | | | 17.736 | | 12 | | .124 | |  |  |
| Linear-by-Linear Association | | | 6.621 | | 1 | | .010 | |  |  |
| N of Valid Cases | | | 42 | |  | |  | |  |  |
| a. 18 cells (85.7%) have expected count less than 5. The minimum expected count is .02. | | | | | | | | |  |  |
| **newAMHcat * No _transfer Crosstabulation** | | | | | | | | | | |
| Count | | | | | | | | | | |
|  | | No _transfer | | | | | | | | Total |
|  |  |  | | 1 | | 2 | | 3 | |  |
| newAMHcat | 3.00 | 0 | | 1 | | 0 | | 0 | | 1 |
|  | 4.00 | 1 | | 4 | | 14 | | 0 | | 19 |
|  | 5.00 | 1 | | 4 | | 14 | | 3 | | 22 |
| Total | | 2 | | 9 | | 28 | | 3 | | 42 |

| **Chi-Square Tests** | | | | | |  |
| --- | --- | --- | --- | --- | --- | --- |
|  | Value | | df | Asymptotic Significance (2-sided) | |  |
| Pearson Chi-Square | 6.615^a^ | | 6 | .358 | |  |
| Likelihood Ratio | 7.112 | | 6 | .311 | |  |
| N of Valid Cases | 42 | |  |  | |  |
|  | | Elapsed Time | | | 00:00:00,00 | |
|  |  | Dimensions Requested | | | 2 | |
|  |  | Cells Available | | | 349496 | |

| **Case Processing Summary** | | | | | | |
| --- | --- | --- | --- | --- | --- | --- |
|  | Cases | | | | | |
|  | Valid | | Missing | | Total | |
|  | N | Percent | N | Percent | N | Percent |
| newAMHcat * Result | 42 | 100.0% | 0 | 0.0% | 42 | 100.0% |

| **newAMHcat * Result Crosstabulation** | | | | |
| --- | --- | --- | --- | --- |
| Count | | | | |
|  | | Result | | Total |
|  |  | Neg | POS |  |
| newAMHcat | 3.00 | 1 | 0 | 1 |
|  | 4.00 | 13 | 6 | 19 |
|  | 5.00 | 16 | 6 | 22 |
| Total | | 30 | 12 | 42 |

| **Chi-Square Tests** | | | |
| --- | --- | --- | --- |
|  | Value | df | Asymptotic Significance (2-sided) |
| Pearson Chi-Square | .502^a^ | 2 | .778 |
| Likelihood Ratio | .774 | 2 | .679 |
| N of Valid Cases | 42 |  |  |
| a. 2 cells (33.3%) have expected count less than 5. The minimum expected count is .29. | | | |

**Crosstabs**

| **Race * Result Crosstabulation** | | | | |
| --- | --- | --- | --- | --- |
| Count | | | | |
|  | | Result | | Total |
|  |  | Neg | POS |  |
| Race | Black | 5 | 3 | 8 |
|  | Colored | 2 | 2 | 4 |
|  | Indian | 17 | 4 | 21 |
|  | White | 6 | 3 | 9 |
| Total | | 30 | 12 | 42 |

| **Chi-Square Tests** | | | |
| --- | --- | --- | --- |
|  | Value | df | Asymptotic Significance (2-sided) |
| Pearson Chi-Square | 2.246^a^ | 3 | .523 |
| Likelihood Ratio | 2.217 | 3 | .529 |
| N of Valid Cases | 42 |  |  |
| a. 4 cells (50.0%) have expected count less than 5. The minimum expected count is 1.14. | | | |
