# Supplementary material for: The relationship between anti-Müllerian hormone (AMH) levels and pregnancy outcomes in patients undergoing assisted reproductive techniques (ART)
Source: PeerJ. 2020 Dec 22;8:e10390. doi: 10.7717/peerj.10390 (PMC7761264; doi:10.7717/peerj.10390)
Supplement: Supplemental Information 1 [file peerj-08-10390-s001.zip › Raw data/Cross tabs on AMH 3cat and No oocytes.docx]

| **AMH3cat * no_oocytesrecode Crosstabulation** | | | | | | | |
| --- | --- | --- | --- | --- | --- | --- | --- |
| Count | | | | | | | |
|  | | no_oocytesrecode | | | | | Total |
|  |  | 1 | 2 | 3 | 4 | 5 or more |  |
| AMH3cat | 1.00 | 1 | 0 | 0 | 0 | 0 | 1 |
|  | 2.00 | 2 | 13 | 2 | 2 | 0 | 19 |
|  | 3.00 | 3 | 3 | 7 | 4 | 5 | 22 |
| Total | | 6 | 16 | 9 | 6 | 5 | 42 |

| **Chi-Square Tests** | | | |
| --- | --- | --- | --- |
|  | Value | df | Asymptotic Significance (2-sided) |
| Pearson Chi-Square | 21.246^a^ | 8 | .007 |
| Likelihood Ratio | 21.317 | 8 | .006 |
| Linear-by-Linear Association | 8.859 | 1 | .003 |
| N of Valid Cases | 42 |  |  |
| a. 13 cells (86.7%) have expected count less than 5. The minimum expected count is .12. | | | |

THIS IS SIGNIFICANT. THERE IS AN ASSOCIATION BETWEEN AMH3cat and no-oocytes.

p-value =0.007
