# Supplementary material for: The relationship between anti-Müllerian hormone (AMH) levels and pregnancy outcomes in patients undergoing assisted reproductive techniques (ART)
Source: PeerJ. 2020 Dec 22;8:e10390. doi: 10.7717/peerj.10390 (PMC7761264; doi:10.7717/peerj.10390)
Supplement: Supplemental Information 1 [file peerj-08-10390-s001.zip › Raw data/logistic regression 27 November.docx]

Logistic regression.

A logistic regression model was fitted to the data with the result of the process namely, “Positive” or “Negative” as the binary dependent variable and Age${, E}_{2}$, LH, Basal FSH, Basal AMH and number of eggs fertilized as the independent variables. The SPSS output for the model is given in Table

| **Variables in the Equation** | | | | | | | |
| --- | --- | --- | --- | --- | --- | --- | --- |
|  | | B | S.E. | Wald | df | Sig. | Exp(B) |
|  | E2 | .001 | .000 | 3.396 | 1 | .065 | 1.001 |
|  | LH | -.556 | .273 | 4.144 | 1 | .042 | .574 |
|  | BasalAMH | -.335 | .239 | 1.967 | 1 | .161 | .715 |
|  | Age | -.146 | .091 | 2.593 | 1 | .107 | .864 |
|  | No_Fertilized | .150 | .368 | .166 | 1 | .683 | 1.162 |
|  | BasalFSH | -.102 | .136 | .559 | 1 | .455 | .903 |
|  | Constant | 4.451 | 3.452 | 1.663 | 1 | .197 | 85.744 |
| As can be seen in the table LH has a p-vlaue =0.042<0.05 and E2 has a p-value of 0.065, which is significant at a 10 % level. | | | | | | | |

The classification table for the model below shows that overall 73.8 % of the cases were correctly classified, while 5/12 =0.417 or 41.7 % of the positives were correctly classified, and 86.7% of the negative cases were correctly classified.

| **Classification Table^a^** | | | | | |
| --- | --- | --- | --- | --- | --- |
|  | outcome | Neg | 26 | 4 | 86.7 |
|  |  | Pos | 7 | 5 | 41.7 |
|  | Overall Percentage | |  |  | 73.8 |
| a. The cut value is .500 | | | | | |

Since No fertilized and Basal FSH have high p-values, they will now be removed from the model and a model with Age, E2, LH and BasalAMH will be fitted.

The model output is summarized in Table ??

| **Variables in the Equation** | | | | | | | |
| --- | --- | --- | --- | --- | --- | --- | --- |
|  | | B | S.E. | Wald | df | Sig. | Exp(B) |
|  | E2 | .001 | .000 | 5.672 | 1 | .017 | 1.001 |
|  | LH | -.571 | .271 | 4.450 | 1 | .035 | .565 |
|  | BasalAMH | -.284 | .224 | 1.607 | 1 | .205 | .753 |
|  | Age | -.146 | .090 | 2.624 | 1 | .105 | .864 |
|  | Constant | 3.680 | 3.100 | 1.409 | 1 | .235 | 39.662 |

In this model E2 has a p-value =0.017<0.05 and LH has a p-value =0.035<0.05. Both of these variables are significant and we keep age and BasalAMH in the model because they play a roll in the pregnancy outcome. The column Exp(B) is the odds ratio.

The interpretation is that an increase of 1000 units in the E2 value will increase the odds of a positive result by exp(1000$\times0.001)$= exp(1) =2.72.

The interpretation for LH is that a 1 unit increase in LH gives a decrease of 0.565 in the odds of a positive result.

| **Classification Table^a^** | | | | | | | | | |
| --- | --- | --- | --- | --- | --- | --- | --- | --- | --- |
| Observed | | | Predicted | | | | | |  |
|  |  |  | outcome | | | | Percentage Correct | |  |
|  |  |  | Neg | | Pos | |  |  |  |
|  | outcome | Neg | | 25 | | 5 | | 83.3 | |
|  |  | Pos | | 7 | | 5 | | 41.7 | |
|  | Overall Percentage | | |  | |  | | 71.4 | |
| a. The cut value is .500 | | | | | | | | | |
