# Supplementary material for: The relationship between anti-Müllerian hormone (AMH) levels and pregnancy outcomes in patients undergoing assisted reproductive techniques (ART)
Source: PeerJ. 2020 Dec 22;8:e10390. doi: 10.7717/peerj.10390 (PMC7761264; doi:10.7717/peerj.10390)
Supplement: Supplemental Information 1 [file peerj-08-10390-s001.zip › Raw data/logistic regression and ROC.docx]

**Regression**

**Crosstabs**

| **No _transfer * Result Crosstabulation** | | | | |
| --- | --- | --- | --- | --- |
| Count | | | | |
|  | | Result | | Total |
|  |  | Neg | POS |  |
| No _transfer |  | 1 | 1 | 2 |
|  | 1 | 7 | 2 | 9 |
|  | 2 | 20 | 8 | 28 |
|  | 3 | 2 | 1 | 3 |
| Total | | 30 | 12 | 42 |

| **Chi-Square Tests** | | | |
| --- | --- | --- | --- |
|  | Value | df | Asymptotic Significance (2-sided) |
| Pearson Chi-Square | .661^a^ | 3 | .882 |
| Likelihood Ratio | .625 | 3 | .891 |
| N of Valid Cases | 42 |  |  |
| a. 5 cells (62,5%) have expected count less than 5. The minimum expected count is ,57. | | | |

| **No_of_oocytes * Result Crosstabulation** | | | | |
| --- | --- | --- | --- | --- |
| Count | | | | |
|  | | Result | | Total |
|  |  | Neg | POS |  |
| No_of_oocytes | 1 | 5 | 1 | 6 |
|  | 2 | 14 | 2 | 16 |
|  | 3 | 5 | 4 | 9 |
|  | 4 | 2 | 4 | 6 |
|  | 5 | 3 | 0 | 3 |
|  | 9 | 1 | 0 | 1 |
|  | 11 | 0 | 1 | 1 |
| Total | | 30 | 12 | 42 |

| **Chi-Square Tests** | | | |
| --- | --- | --- | --- |
|  | Value | df | Asymptotic Significance (2-sided) |
| Pearson Chi-Square | 11.919^a^ | 6 | .064 |
| Likelihood Ratio | 12.788 | 6 | .047 |
| N of Valid Cases | 42 |  |  |
| a. 12 cells (85,7%) have expected count less than 5. The minimum expected count is ,29. | | | |

| **no_oocytesrecode * Result Crosstabulation** | | | | |
| --- | --- | --- | --- | --- |
| Count | | | | |
|  | | Result | | Total |
|  |  | Neg | POS |  |
| no_oocytesrecode | 1.00 | 5 | 1 | 6 |
|  | 2.00 | 14 | 2 | 16 |
|  | 3.00 | 5 | 4 | 9 |
|  | 4.00 | 6 | 5 | 11 |
| Total | | 30 | 12 | 42 |

| **Chi-Square Tests** | | | |
| --- | --- | --- | --- |
|  | Value | df | Asymptotic Significance (2-sided) |
| Pearson Chi-Square | 5.089^a^ | 3 | .165 |
| Likelihood Ratio | 5.268 | 3 | .153 |
| N of Valid Cases | 42 |  |  |
| a. 5 cells (62,5%) have expected count less than 5. The minimum expected count is 1,71. | | | |

| **no_oocytesrecode * Result Crosstabulation** | | | | |
| --- | --- | --- | --- | --- |
| Count | | | | |
|  | | Result | | Total |
|  |  | Neg | POS |  |
| no_oocytesrecode | 1.00 | 5 | 1 | 6 |
|  | 2.00 | 14 | 2 | 16 |
|  | 3.00 | 5 | 4 | 9 |
|  | 4.00 | 2 | 4 | 6 |
|  | 5.00 | 4 | 1 | 5 |
| Total | | 30 | 12 | 42 |

| **Chi-Square Tests** | | | |
| --- | --- | --- | --- |
|  | Value | df | Asymptotic Significance (2-sided) |
| Pearson Chi-Square | 7.999^a^ | 4 | .092 |
| Likelihood Ratio | 7.784 | 4 | .100 |
| N of Valid Cases | 42 |  |  |
| a. 8 cells (80,0%) have expected count less than 5. The minimum expected count is 1,43. | | | |

|  |
| --- |

**Block 1: Method = Enter**

| **Classification Table^a^** | | | | | |
| --- | --- | --- | --- | --- | --- |
|  | Observed | | Predicted | | |
|  |  |  | Result | | Percentage Correct |
|  |  |  | Neg | POS |  |
| Step 1 | Result | Neg | 26 | 4 | 86.7 |
|  |  | POS | 7 | 5 | 41.7 |
|  | Overall Percentage | |  |  | 73.8 |
| a. The cut value is ,500 | | | | | |

| **Variables in the Equation** | | | | | | | |
| --- | --- | --- | --- | --- | --- | --- | --- |
|  | | B | S.E. | Wald | df | Sig. | Exp(B) |
| Step 1^a^ | Age | -.146 | .091 | 2.593 | 1 | .107 | .864 |
|  | E2 | .001 | .000 | 3.396 | 1 | .065 | 1.001 |
|  | LH | -.556 | .273 | 4.144 | 1 | .042 | .574 |
|  | BasalAMH | -.335 | .239 | 1.967 | 1 | .161 | .715 |
|  | BasalFSH | -.102 | .136 | .559 | 1 | .455 | .903 |
|  | No_Fertilized | .150 | .368 | .166 | 1 | .683 | 1.162 |
|  | Constant | 4.451 | 3.452 | 1.663 | 1 | .197 | 85.744 |
| a. Variable(s) entered on step 1: Age, E2, LH, BasalAMH, BasalFSH, No_Fertilized. | | | | | | | |

**Logistic Regression**

**Block 1: Method = Enter**

| **Model Summary** | | | |
| --- | --- | --- | --- |
| Step | -2 Log likelihood | Cox & Snell R Square | Nagelkerke R Square |
| 1 | 37.574^a^ | .261 | .373 |
| a. Estimation terminated at iteration number 6 because parameter estimates changed by less than ,001. | | | |

| **Classification Table^a^** | | | | | |
| --- | --- | --- | --- | --- | --- |
|  | Observed | | Predicted | | |
|  |  |  | Result | | Percentage Correct |
|  |  |  | Neg | POS |  |
| Step 1 | Result | Neg | 25 | 5 | 83.3 |
|  |  | POS | 8 | 4 | 33.3 |
|  | Overall Percentage | |  |  | 69.0 |
| a. The cut value is ,500 | | | | | |

| **Variables in the Equation** | | | | | | | |
| --- | --- | --- | --- | --- | --- | --- | --- |
|  | | B | S.E. | Wald | df | Sig. | Exp(B) |
| Step 1^a^ | Age | -.149 | .090 | 2.730 | 1 | .098 | .862 |
|  | E2 | .001 | .000 | 4.992 | 1 | .025 | 1.001 |
|  | LH | -.549 | .262 | 4.396 | 1 | .036 | .577 |
|  | BasalAMH | -.317 | .233 | 1.846 | 1 | .174 | .729 |
|  | BasalFSH | -.087 | .129 | .453 | 1 | .501 | .917 |
|  | Constant | 4.613 | 3.421 | 1.818 | 1 | .178 | 100.794 |
| a. Variable(s) entered on step 1: Age, E2, LH, BasalAMH, BasalFSH. | | | | | | | |

**Logistic Regression**

| **Variables in the Equation** | | | | | | | |
| --- | --- | --- | --- | --- | --- | --- | --- |
|  | | B | S.E. | Wald | df | Sig. | Exp(B) |
| Step 0 | Constant | -.916 | .342 | 7.196 | 1 | .007 | .400 |

| **Variables not in the Equation** | | | | | |
| --- | --- | --- | --- | --- | --- |
|  | | | Score | df | Sig. |
| Step 0 | Variables | Age | 1.760 | 1 | .185 |
|  |  | E2 | 3.985 | 1 | .046 |
|  |  | LH | 1.400 | 1 | .237 |
|  |  | BasalAMH | .016 | 1 | .900 |
|  | Overall Statistics | | 9.453 | 4 | .051 |

**Block 1: Method = Enter**

| **Model Summary** | | | |
| --- | --- | --- | --- |
| Step | -2 Log likelihood | Cox & Snell R Square | Nagelkerke R Square |
| 1 | 38.053^a^ | .252 | .361 |
| a. Estimation terminated at iteration number 6 because parameter estimates changed by less than ,001. | | | |

| **Classification Table^a^** | | | | | |
| --- | --- | --- | --- | --- | --- |
|  | Observed | | Predicted | | |
|  |  |  | Result | | Percentage Correct |
|  |  |  | Neg | POS |  |
| Step 1 | Result | Neg | 25 | 5 | 83.3 |
|  |  | POS | 7 | 5 | 41.7 |
|  | Overall Percentage | |  |  | 71.4 |
| a. The cut value is ,500 | | | | | |

| **Variables in the Equation** | | | | | | | |
| --- | --- | --- | --- | --- | --- | --- | --- |
|  | | B | S.E. | Wald | df | Sig. | Exp(B) |
| Step 1^a^ | Age | -.146 | .090 | 2.624 | 1 | .105 | .864 |
|  | E2 | .001 | .000 | 5.672 | 1 | .017 | 1.001 |
|  | LH | -.571 | .271 | 4.450 | 1 | .035 | .565 |
|  | BasalAMH | -.284 | .224 | 1.607 | 1 | .205 | .753 |
|  | Constant | 3.680 | 3.100 | 1.409 | 1 | .235 | 39.662 |
| a. Variable(s) entered on step 1: Age, E2, LH, BasalAMH. | | | | | | | |

**Logistic Regression**

| **Variables in the Equation** | | | | | | | |
| --- | --- | --- | --- | --- | --- | --- | --- |
|  | | B | S.E. | Wald | df | Sig. | Exp(B) |
| Step 0 | Constant | -.916 | .342 | 7.196 | 1 | .007 | .400 |

| **Variables not in the Equation** | | | | | |
| --- | --- | --- | --- | --- | --- |
|  | | | Score | df | Sig. |
| Step 0 | Variables | Age | 1.760 | 1 | .185 |
|  |  | E2 | 3.985 | 1 | .046 |
|  |  | LH | 1.400 | 1 | .237 |
|  |  | BasalAMH | .016 | 1 | .900 |
|  |  | No_mature | 3.734 | 1 | .053 |
|  | Overall Statistics | | 10.022 | 5 | .075 |

**Block 1: Method = Enter**

| **Model Summary** | | | |
| --- | --- | --- | --- |
| Step | -2 Log likelihood | Cox & Snell R Square | Nagelkerke R Square |
| 1 | 37.280^a^ | .266 | .381 |
| a. Estimation terminated at iteration number 6 because parameter estimates changed by less than ,001. | | | |

| **Classification Table^a^** | | | | | |
| --- | --- | --- | --- | --- | --- |
|  | Observed | | Predicted | | |
|  |  |  | Result | | Percentage Correct |
|  |  |  | Neg | POS |  |
| Step 1 | Result | Neg | 26 | 4 | 86.7 |
|  |  | POS | 8 | 4 | 33.3 |
|  | Overall Percentage | |  |  | 71.4 |
| a. The cut value is ,500 | | | | | |

| **Variables in the Equation** | | | | | | | |
| --- | --- | --- | --- | --- | --- | --- | --- |
|  | | B | S.E. | Wald | df | Sig. | Exp(B) |
| Step 1^a^ | Age | -.142 | .091 | 2.451 | 1 | .117 | .867 |
|  | E2 | .001 | .000 | 3.580 | 1 | .058 | 1.001 |
|  | LH | -.600 | .303 | 3.923 | 1 | .048 | .549 |
|  | BasalAMH | -.309 | .229 | 1.821 | 1 | .177 | .734 |
|  | No_mature | .261 | .304 | .737 | 1 | .390 | 1.298 |
|  | Constant | 3.333 | 3.156 | 1.116 | 1 | .291 | 28.031 |
| a. Variable(s) entered on step 1: Age, E2, LH, BasalAMH, No_mature. | | | | | | | |

**Logistic Regression**

| **Classification Table^a,b^** | | | | | |
| --- | --- | --- | --- | --- | --- |
|  | Observed | | Predicted | | |
|  |  |  | Result | | Percentage Correct |
|  |  |  | Neg | POS |  |
| Step 0 | Result | Neg | 30 | 0 | 100.0 |
|  |  | POS | 12 | 0 | .0 |
|  | Overall Percentage | |  |  | 71.4 |
| a. Constant is included in the model. | | | | | |
| b. The cut value is ,500 | | | | | |

| **Variables in the Equation** | | | | | | | |
| --- | --- | --- | --- | --- | --- | --- | --- |
|  | | B | S.E. | Wald | df | Sig. | Exp(B) |
| Step 0 | Constant | -.916 | .342 | 7.196 | 1 | .007 | .400 |

| **Variables not in the Equation** | | | | | |
| --- | --- | --- | --- | --- | --- |
|  | | | Score | df | Sig. |
| Step 0 | Variables | Age | 1.760 | 1 | .185 |
|  |  | BasalFSH | .735 | 1 | .391 |
|  |  | BasalAMH | .016 | 1 | .900 |
|  | Overall Statistics | | 2.840 | 3 | .417 |

**Block 1: Method = Enter**

| **Omnibus Tests of Model Coefficients** | | | | |
| --- | --- | --- | --- | --- |
|  | | Chi-square | df | Sig. |
| Step 1 | Step | 2.973 | 3 | .396 |
|  | Block | 2.973 | 3 | .396 |
|  | Model | 2.973 | 3 | .396 |

| **Model Summary** | | | |
| --- | --- | --- | --- |
| Step | -2 Log likelihood | Cox & Snell R Square | Nagelkerke R Square |
| 1 | 47.282^a^ | .068 | .098 |
| a. Estimation terminated at iteration number 4 because parameter estimates changed by less than ,001. | | | |

| **Classification Table^a^** | | | | | |
| --- | --- | --- | --- | --- | --- |
|  | Observed | | Predicted | | |
|  |  |  | Result | | Percentage Correct |
|  |  |  | Neg | POS |  |
| Step 1 | Result | Neg | 29 | 1 | 96.7 |
|  |  | POS | 10 | 2 | 16.7 |
|  | Overall Percentage | |  |  | 73.8 |
| a. The cut value is ,500 | | | | | |

| **Variables in the Equation** | | | | | | | |
| --- | --- | --- | --- | --- | --- | --- | --- |
|  | | B | S.E. | Wald | df | Sig. | Exp(B) |
| Step 1^a^ | Age | -.106 | .076 | 1.959 | 1 | .162 | .900 |
|  | BasalFSH | -.111 | .109 | 1.036 | 1 | .309 | .895 |
|  | BasalAMH | -.061 | .183 | .111 | 1 | .739 | .941 |
|  | Constant | 3.533 | 2.798 | 1.594 | 1 | .207 | 34.214 |
| a. Variable(s) entered on step 1: Age, BasalFSH, BasalAMH. | | | | | | | |

**Logistic Regression**

| **Variables in the Equation** | | | | | | | |
| --- | --- | --- | --- | --- | --- | --- | --- |
|  | | B | S.E. | Wald | df | Sig. | Exp(B) |
| Step 0 | Constant | -.916 | .342 | 7.196 | 1 | .007 | .400 |

| **Variables not in the Equation** | | | | | |
| --- | --- | --- | --- | --- | --- |
|  | | | Score | df | Sig. |
| Step 0 | Variables | Age | 1.760 | 1 | .185 |
|  |  | BasalFSH | .735 | 1 | .391 |
|  |  | BasalAMH | .016 | 1 | .900 |
|  |  | E2 | 3.985 | 1 | .046 |
|  |  | LH | 1.400 | 1 | .237 |
|  |  | No_Fertilized | 2.473 | 1 | .116 |
|  | Overall Statistics | | 10.420 | 6 | .108 |

**Block 1: Method = Enter**

| **Model Summary** | | | |
| --- | --- | --- | --- |
| Step | -2 Log likelihood | Cox & Snell R Square | Nagelkerke R Square |
| 1 | 37.407^a^ | .264 | .378 |
| a. Estimation terminated at iteration number 6 because parameter estimates changed by less than ,001. | | | |

| **Classification Table^a^** | | | | | |
| --- | --- | --- | --- | --- | --- |
|  | Observed | | Predicted | | |
|  |  |  | Result | | Percentage Correct |
|  |  |  | Neg | POS |  |
| Step 1 | Result | Neg | 26 | 4 | 86.7 |
|  |  | POS | 7 | 5 | 41.7 |
|  | Overall Percentage | |  |  | 73.8 |
| a. The cut value is ,500 | | | | | |

| **Variables in the Equation** | | | | | | | |
| --- | --- | --- | --- | --- | --- | --- | --- |
|  | | B | S.E. | Wald | df | Sig. | Exp(B) |
| Step 1^a^ | Age | -.146 | .091 | 2.593 | 1 | .107 | .864 |
|  | BasalFSH | -.102 | .136 | .559 | 1 | .455 | .903 |
|  | BasalAMH | -.335 | .239 | 1.967 | 1 | .161 | .715 |
|  | E2 | .001 | .000 | 3.396 | 1 | .065 | 1.001 |
|  | LH | -.556 | .273 | 4.144 | 1 | .042 | .574 |
|  | No_Fertilized | .150 | .368 | .166 | 1 | .683 | 1.162 |
|  | Constant | 4.451 | 3.452 | 1.663 | 1 | .197 | 85.744 |
| a. Variable(s) entered on step 1: Age, BasalFSH, BasalAMH, E2, LH, No_Fertilized. | | | | | | | |

**ROC Curve**

| **Case Processing Summary** | |
| --- | --- |
| outcome | Valid N (listwise) |
| Positive^a^ | 12 |
| Negative | 30 |
| Larger values of the test result variable(s) indicate stronger evidence for a positive actual state. | |
| a. The positive actual state is 1,00. | |


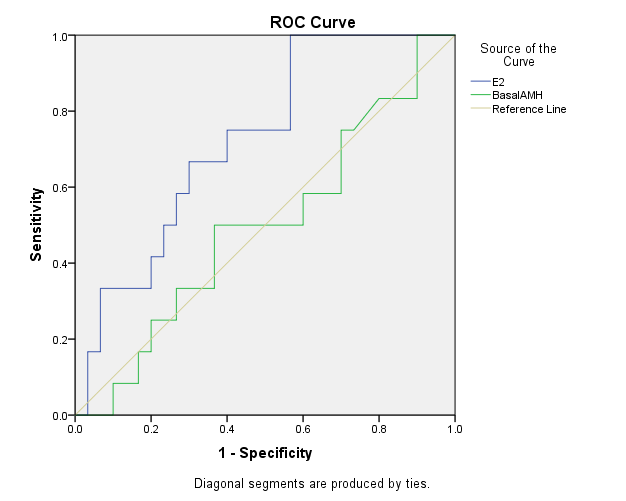


| **Area Under the Curve** | |
| --- | --- |
| Test Result Variable(s) | Area |
| E2 | .725 |
| BasalAMH | .497 |
| The test result variable(s): BasalAMH has at least one tie between the positive actual state group and the negative actual state group. Statistics may be biased. | |

**Logistic Regression**

| **Dependent Variable Encoding** | |
| --- | --- |
| Original Value | Internal Value |
| Neg | 0 |
| POS | 1 |

| **Classification Table^a,b^** | | | | | |
| --- | --- | --- | --- | --- | --- |
|  | Observed | | Predicted | | |
|  |  |  | Result | | Percentage Correct |
|  |  |  | Neg | POS |  |
| Step 0 | Result | Neg | 30 | 0 | 100.0 |
|  |  | POS | 12 | 0 | .0 |
|  | Overall Percentage | |  |  | 71.4 |
| a. Constant is included in the model. | | | | | |
| b. The cut value is ,500 | | | | | |

| **Variables in the Equation** | | | | | | | |
| --- | --- | --- | --- | --- | --- | --- | --- |
|  | | B | S.E. | Wald | df | Sig. | Exp(B) |
| Step 0 | Constant | -.916 | .342 | 7.196 | 1 | .007 | .400 |

| **Variables not in the Equation** | | | | | |
| --- | --- | --- | --- | --- | --- |
|  | | | Score | df | Sig. |
| Step 0 | Variables | Age | 1.760 | 1 | .185 |
|  |  | BasalFSH | .735 | 1 | .391 |
|  |  | BasalAMH | .016 | 1 | .900 |
|  |  | E2 | 3.985 | 1 | .046 |
|  |  | LH | 1.400 | 1 | .237 |
|  |  | No_Fertilized | 2.473 | 1 | .116 |
|  | Overall Statistics | | 10.420 | 6 | .108 |

**Block 1: Method = Enter**

| **Omnibus Tests of Model Coefficients** | | | | |
| --- | --- | --- | --- | --- |
|  | | Chi-square | df | Sig. |
| Step 1 | Step | 12.848 | 6 | .046 |
|  | Block | 12.848 | 6 | .046 |
|  | Model | 12.848 | 6 | .046 |

| **Model Summary** | | | |
| --- | --- | --- | --- |
| Step | -2 Log likelihood | Cox & Snell R Square | Nagelkerke R Square |
| 1 | 37.407^a^ | .264 | .378 |
| a. Estimation terminated at iteration number 6 because parameter estimates changed by less than ,001. | | | |

| **Classification Table^a^** | | | | | |
| --- | --- | --- | --- | --- | --- |
|  | Observed | | Predicted | | |
|  |  |  | Result | | Percentage Correct |
|  |  |  | Neg | POS |  |
| Step 1 | Result | Neg | 26 | 4 | 86.7 |
|  |  | POS | 7 | 5 | 41.7 |
|  | Overall Percentage | |  |  | 73.8 |
| a. The cut value is ,500 | | | | | |

| **Variables in the Equation** | | | | | | | |
| --- | --- | --- | --- | --- | --- | --- | --- |
|  | | B | S.E. | Wald | df | Sig. | Exp(B) |
| Step 1^a^ | Age | -.146 | .091 | 2.593 | 1 | .107 | .864 |
|  | BasalFSH | -.102 | .136 | .559 | 1 | .455 | .903 |
|  | BasalAMH | -.335 | .239 | 1.967 | 1 | .161 | .715 |
|  | E2 | .001 | .000 | 3.396 | 1 | .065 | 1.001 |
|  | LH | -.556 | .273 | 4.144 | 1 | .042 | .574 |
|  | No_Fertilized | .150 | .368 | .166 | 1 | .683 | 1.162 |
|  | Constant | 4.451 | 3.452 | 1.663 | 1 | .197 | 85.744 |
| a. Variable(s) entered on step 1: Age, BasalFSH, BasalAMH, E2, LH, No_Fertilized. | | | | | | | |

**Crosstabs**

| **no_mature_cat * Result Crosstabulation** | | | | |
| --- | --- | --- | --- | --- |
| Count | | | | |
|  | | Result | | Total |
|  |  | Neg | POS |  |
| no_mature_cat | .00 | 4 | 0 | 4 |
|  | 1.00 | 5 | 1 | 6 |
|  | 2.00 | 12 | 2 | 14 |
|  | 3.00 | 4 | 5 | 9 |
|  | 4.00 | 5 | 4 | 9 |
| Total | | 30 | 12 | 42 |

| **Chi-Square Tests** | | | |
| --- | --- | --- | --- |
|  | Value | df | Asymptotic Significance (2-sided) |
| Pearson Chi-Square | 7.739^a^ | 4 | .102 |
| Likelihood Ratio | 8.634 | 4 | .071 |
| N of Valid Cases | 42 |  |  |
| a. 7 cells (70,0%) have expected count less than 5. The minimum expected count is 1,14. | | | |


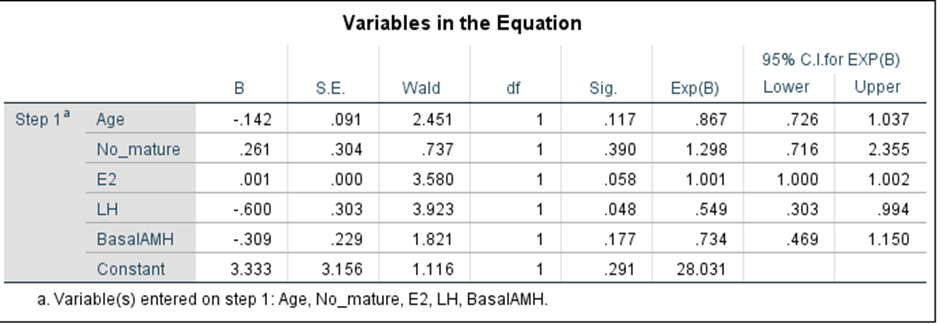


The table below was the best logistic regression model showing the E2 and LH are significant and not the AMH.
