# Supplementary figures and images for: The relationship between anti-Müllerian hormone (AMH) levels and pregnancy outcomes in patients undergoing assisted reproductive techniques (ART)
Source: PeerJ. 2020 Dec 22;8:e10390. doi: 10.7717/peerj.10390 (PMC7761264; doi:10.7717/peerj.10390)

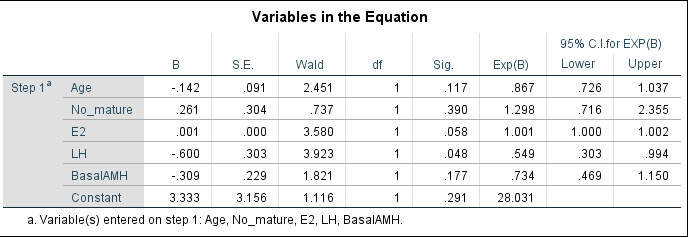

Supplement: Supplemental Information 1 [file peerj-08-10390-s001.zip › Raw data/Variables.png]
