# Supplementary material for: The relationship between anti-Müllerian hormone (AMH) levels and pregnancy outcomes in patients undergoing assisted reproductive techniques (ART)
Source: PeerJ. 2020 Dec 22;8:e10390. doi: 10.7717/peerj.10390 (PMC7761264; doi:10.7717/peerj.10390)
Supplement: Supplemental Information 2 [file peerj-08-10390-s002.docx]

S1 Table 1: Prediction of pregnancy outcome by SPSS software

| Observed | | Predicted | | |
| --- | --- | --- | --- | --- |
|  |  |  | | Percentage Correct |
|  |  | Negative | Positive |  |
| outcome | Negative | 26 | 4 | 86.7 |
|  | Positive | 7 | 5 | 41.7 |
| Overall Percentage | |  |  | 73.8 |
